# Supplementary material for: Review of Methodological Approaches to Human Milk Small Extracellular Vesicle Proteomics
Source: Biomolecules. 2021 Jun 3;11(6):833. doi: 10.3390/biom11060833 (PMC8228857; doi:10.3390/biom11060833)
Supplement: Supplementary file 1 [file biomolecules-11-00833-s001.zip › biomolecules-1226406-supplementary.pdf]

Supplementary tables

Table S1. Summary of published sample preparations for proteomics analyses of human milk extracellular vesicles.

Reference	Proteomics sample preparation	End-goal	Nr of proteins detected
(Admyre *et al.*, 2007)	1) 50 µg of exosomes in denaturing buffer (6 M urea, 0.05% SDS, 5 mM EDTA, and 50 mM Tris-HCl (pH 8.5))<br>2) Digestion with 2 µg of trypsin overnight at 37°C.<br>3) Peptide elution using cation exchange columns.<br>4) Peptide purification using C18 Empore membrane (3M).<br>*Tandem MS (MS/MS) analysis:*<br>1) Peptide separation using nano-HPLC with a linear gradient (2% ACN to 95% ACN).<br>2) Peptides sent to ESI-QqTOF mass spectrometer.<br>3) Data collected at MS1 (first 1s of data) with two most intense peaks for MS2 (first 3s of data).	Characterization of exosomes	73
(Van Herwijnen *et al.*, 2016)	1) 20 µl or 40 µl of sonicated EVs added to 10 µl 200 mm DTT and 10 µl sample buffer.<br>2) Samples separated on a 12% Tris-HCl gel.<br>3) In gel digestion using trypsin.<br>*Tandem MS (MS/MS) analysis:*<br>1a) Peptide separation using Proxeon EASY-nLC 1000 with a C18 trap column.<br>2a) Peptides sent to LTQ-Orbitrap Elite.<br>3a) Data collected at MS1 at selected survey scan of 350 to 1500 mass/charge (m/z) with ten most abundant precursors for MS2 fragmentation.<br>1b) Peptide separation using Agilent 1290 Infinity LC system with a C18 trap column.<br>2b) Peptides sent to Q-Exactive instrument.<br>3b) Data collected at MS1 at selected survey scan of 350 to 1500 mass/charge (m/z) with ten most abundant precursors for MS2 fragmentation.	Proteomic characterization of vesicles	1963
(Yang *et al.*, 2017)	1) 200 µg of exosomes in denaturing buffer (4% SDS, 150 mM Tris-HCl pH 8.0, 100 mM DTT).<br>2) Exosomes mixed with UT buffer (8 M urea and 150 mM Tris-HCl at pH 8.0) and 0.05 M	Proteomic comparison of bovine and human milk samples	920

	iodoacetamide solution, washed several times with UT buffer.<br>3) Digestion with 40 µL trypsin at 37 °C for 24 h.<br>4) Obtained peptides labeled with iTRAQ reagents (Reagent-8plex Multiplex Kit)<br>5) Peptide separation using strong cation exchange (SCX) chromatography.<br>6) Sample desalted using C18 solid phase extraction column.<br>*Tandem MS (MS/MS) analysis:*<br>1) Peptide separation using Easy-nLC.<br>2) Peptides sent to Q-Exactive mass spectrometer.<br>3) Data collected at MS1 at selected survey scan of 300–1800 mass/charge (m/z) with ten most abundant precursors for MS2 data at 2 m/z isolation window.		
(Wang *et al.*, 2019)	1) Exosomes in U2 buffer (8 m Urea,100 mm TEAB, pH 8.0) , then reduced with 10 mm DTT and alkylated with 50 mm iodoacetamide<br>2) Peptides larger than 10kDa removed by filtration.<br>3) Peptides purified by C18 columns.<br>4) Peptides labeled with iTRAQ reagents (iTRAQ 8-plex kit).<br>5) Sample fractionation using HPLC.<br>*Tandem MS (MS/MS) analysis:*<br>1) Peptides separated using Eksigent nLC.<br>2) Peptides sent Triple TOF 5600 Plus mass spectrometer.<br>3) Data collected at MS1 selected range of 350-1500 m/z, MS2 spectra at 100-1500 m/z.	Characterization of human term and preterm milk exosomes	719

Table S2. Summary of advantages (+) and disadvantages (-) of methods recommended for human milk extracellular proteomics.

**Isolation**	**Differential ultra-centrifugation**		**Sucrose gradient**	
	+ Separation of EVs without added solutions<br>+ Increased yield	- Time-consuming<br>- Multiple steps<br>- Specialized equipment	+ Increased EV purity<br>+ Size separation in EV populations	- Additional reagents<br>- Time-consuming
**Protein solubilization and peptide cleavage**	**SDS with/out EDTA**		**Trypsin/LysC**	
	+ Effective protein solubilization<br>+ Addition of EDTA minimizes damage to MS equipment	- Reduced compatibility with alternative peptide fractionations, such as in-gel peptide separation	+ Cleaves lysine and arginine bonds<br>+ Increases peptide detection in MS/MS	- No cleavage of serine or threonine<br>- Time-consuming
**Peptide fractionation**	**Reverse phase nano-liquid chromatography**			
	+ Less sample, consumables required<br>+ Increased peptide selectivity and sensitivity<br>+ Easy coupling to MS/MS equipment<br>+ Faster analysis		- Cost<br>- Equipment accessibility	

**Peptide detection**	**Tandem mass spectrometry (MS/MS)**			
	+ Increased protein sensitivity, identification<br>+ Moderate to fast analysis<br>+ Easy coupling to nano-LC equipment		- Difficulty resolving complex samples<br>- Sensitive to buffer salts, contaminants<br>- Incompatible with certain solvents<br>- Cost	
**Data acquisition**	**Data dependent acquisition**		**Data independent acquisition**	
	+ Straight-forward analysis<br>+ Cost<br>+ Increased sensitivity for quantification	- Reduced identification of low abundance peptides<br>- Reduced fragmentation precision	+ Increased peptide detection/identification<br>+Identification of low abundance peptides	- Increased peptide fragmentation<br>- Increased MS/MS spectra complexity
**Data quantification**	**Label-free**		**Isobaric label**	
	+ Automation of analysis<br>+ Unlimited number of quantitative comparisons	- Reduced accuracy for quantification	+ Up to 100% labelling efficiency<br>+ Increased accuracy of quantification	- Limited number of biological samples<br>- Cost
